# A facile solid-state heating method for preparation of poly(3,4-ethelenedioxythiophene)/ZnO nanocomposite and photocatalytic activity

**DOI:** 10.1186/1556-276X-9-89

**Published:** 2014-02-20

**Authors:** Tursun Abdiryim, Ahmat Ali, Ruxangul Jamal, Yakupjan Osman, Yu Zhang

**Affiliations:** 1 Key Laboratory of Petroleum and Gas Fine Chemicals, Educational Ministry of China, School of Chemistry and Chemical Engineering, Xinjiang University, Urumqi 830046, People's Republic of China; 2 Key Laboratory of Functional Polymers, Xinjiang University, Urumqi 830046, People's Republic of China; 3 Key Laboratory of Oil and Gas Fine Chemicals, Educational Ministry of China, College of Chemistry and Chemical Engineering, Xinjiang University, Urumqi 830046, People's Republic of China

**Keywords:** Solid-state heating method, Poly(3,4-ethylenedioxythiophene), Nano-ZnO, Composite, Photocatalyst

## Abstract

Poly(3,4-ethylenedioxythiophene)/zinc oxide (PEDOT/ZnO) nanocomposites were prepared by a simple solid-state heating method, in which the content of ZnO was varied from 10 to 20 wt%. The structure and morphology of the composites were characterized by Fourier transform infrared (FTIR) spectroscopy, ultraviolet-visible (UV-vis) absorption spectroscopy, X-ray diffraction (XRD), and transmission electron microscopy (TEM). The photocatalytic activities of the composites were investigated by the degradation of methylene blue (MB) dye in aqueous medium under UV light and natural sunlight irradiation. The FTIR, UV-vis, and XRD results showed that the composites were successfully synthesized, and there was a strong interaction between PEDOT and nano-ZnO. The TEM results suggested that the composites were a mixture of shale-like PEDOT and less aggregated nano-ZnO. The photocatalytic activity results indicated that the incorporation of ZnO nanoparticles in composites can enhance the photocatalytic efficiency of the composites under both UV light and natural sunlight irradiation, and the highest photocatalytic efficiency under UV light (98.7%) and natural sunlight (96.6%) after 5 h occurred in the PEDOT/15wt%ZnO nanocomposite.

## Background

In recent years, there has been an increasing interest in the development of polymer/inorganic nanohybrid materials [1-3]. Inorganic semiconductors such as ZnO, TiO_2_, MnO_2_, and ZrO_2_ have been extensively investigated as hybrids with polymers having synergetic or complementary properties and behavior for the fabrication of a variety of devices. Among these semiconductors, ZnO has promising applications in electrical engineering, catalysis, ultraviolet absorption, photodegradation of microorganisms, and optical and optoelectronic devices [4-8]. Although ZnO exhibits many advantages, there are still some disadvantages such as the lack of visible light response, low quantum yield, and lower photocatalytic activity. Also, it is important to shift the photoactivation region of ZnO particles toward visible wavelengths. Previous studies demonstrated that conducting polymers incorporated with ZnO could display reasonable catalytic activity under light illumination [9-12], and the delocalized conjugated structures of conductive polymers have been proven to arouse a rapid photoinduced charge separation and decrease the charge recombination rate in electron transfer processes [13,14].

However, ZnO is an amphoteric oxide, and it can react with acid or base to form a water-soluble salt. Therefore, a successful incorporation of ZnO into a conducting polymer matrix is the main research topic. Up to now, there are many reports on the preparation methods of conducting polymer/ZnO composites [15-17], and the methods are mainly electrochemical polymerization [18] and mechanical mixing [19]. Since ZnO has the possibility of forming a soluble salt, the common chemical oxidative polymerization method is difficult to apply for preparing conducting polymer/ZnO composites. Although electrochemical polymerization can be an effective method for obtaining conducting polymer/ZnO composites, the composites are just the layer-by-layer hybrid films of conducting polymers and ZnO, which is the main factor in limiting the use of the composites. In mechanical mixing method, the composites were just the physical mixture of inorganic particles and polymer, and the polymer should be prepared before the mechanochemical mixing [20,21]. The uniform distribution of inorganic particles in the polymer matrix is considered to be difficult in the case of mechanical mixing method.

Among conducting polymers, polyaniline and polythiophene are widely used for the fabrication of conducting polymer/ZnO hybrid materials [22,23]. Although there are many reports about polythiophene-type conducting polymer/ZnO nanohybrid materials, the main aspect of these studies is on the investigation of hybrid bulk heterojunction solar cells based on the blend of polythiophene-type conducting polymers and ZnO nanoparticles [24-26]. As a derivative of polythiophene, poly(3,4-ethylenedioxythiophene) (PEDOT) has been utilized as a charge storage material because of its many favorable properties, including reduced bandgap, low oxidation potential for conversion to the conducting state, and high stability in the conducting form, as well as its larger electroactive potential window and higher cycling stability than polyaniline [27-29]. Sharma et al. reported that PEDOT/ZnO nanocomposite films displayed improved *I*-*V* characteristics, indicating that the heterojunction of nano-ZnO and PEDOT can enhance their photovoltaic properties [30]. Zhang et al. fabricated a self-powered UV photodetector on the basis of the property of the PEDOT:PSS/ZnO heterojunction, which may offer theoretical support in future optoelectronic device fabrication and modification [31]. However, up to now, there is no report for the application of PEDOT/ZnO for dye ultraviolet-visible (UV-vis) photodegradation.

According to the previous report, PEDOT can be prepared by *in situ* sublimation/polymerization of 2,5-dibromo-EDOT [32]. This may bring some possibility of the preparation of a PEDOT/ZnO nanohybrid material by the same method. Herein, we report the exploration of synthesizing PEDOT/ZnO nanocomposites in powder form by *in situ* solid-state heating method, and the content of nano-ZnO in the reaction system was varied from 10 to 20 wt%. The structural and morphological properties of the composites were investigated by Fourier transform infrared (FTIR) spectroscopy, UV-vis absorption spectroscopy, X-ray diffraction (XRD), and transmission electron microscopy (TEM). Furthermore, the comparative photocatalytic activity of the PEDOT/ZnO nanocomposites, nano-ZnO, as well as PEDOT under different light sources for the degradation of methylene blue (MB) was investigated.

## Methods

### Materials

3,4-Ethylenedioxythiophene (EDOT) was obtained from Shanghai Aladdin Reagent Company (Shanghai, China), and it was purified by distillation under reduced pressure and stored in a refrigerator prior to use. Nano-ZnO (with an average diameter of 50 nm) and a silane coupling agent to modify nano-ZnO, KH-540 (γ-aminopropyltrimethoxysilane), were provided by Shanghai Aladdin Reagent Company. All other reagents were of analytical grade and used as supplied without further purification.

### Synthesis of 2,5-dibromo-EDOT

2,5-Dibromo-EDOT (2,5-dibromo-3,4-ethylenedioxythiophene) was synthesized according to the previous report [33].

### Surface modification of nano-ZnO

According to the literature [34], nano-ZnO was exposed to ambient atmosphere for 24 h to generate high-density Zn-OH groups on its surface, followed by drying at 120°C for 2 h. Then, it was immersed in a solution of the silane coupling agent KH-540 (γ-aminopropyltrimethoxysilane) in ethanol (1 g in 100 mL of ethanol) under stirring at 80°C for 10 h and washed with ethanol in ultrasonic bath. Finally, the solution was filtered and dried for further use.

### Synthesis of the PEDOT/ZnO nanocomposites

A mixture of 0.56 g (2 mmol) 2,5-dibromo-EDOT (2,5-dibromo-3,4-ethylenedioxythiophene) and 0.056 g modified nano-ZnO in 30 mL chloroform was ultrasonicated for 30 min to facilitate the monomer to adsorb on the surface of the nano-ZnO. After ultrasonication, the mixture was placed in a vacuum oven at 60°C to evaporate the chloroform, and then the residue was kept in a vacuum oven under the same conditions for 24 h. The obtained composite was denoted as PEDOT/10wt%ZnO. The PEDOT/15wt%ZnO and PEDOT/20wt%ZnO composites were prepared in a similar manner by adjusting the weight percentage of the nano-ZnO in the reaction medium as 15% and 20%, respectively. For comparison, the pure PEDOT was also synthesized in a similar manner without adding the nano-ZnO in the reaction medium.

### Characterization techniques

The FTIR spectra of the composites were obtained using a BRUKER EQUINOX-55 Fourier transform infrared spectrometer (Bruker, Billerica, MA, USA) (frequency range 4,000 to 500 cm^-1^). The UV-vis spectra of the samples were recorded on a UV-vis spectrophotometer (UV4802, Unico, Dayton, NJ, USA). XRD patterns have been obtained using a Bruker AXS D8 diffractometer with a monochromatic Cu-Kα radiation source (*λ* = 0.15418 nm); the scan range (2*θ*) was 5° to 70°. TEM measurements were performed on a TEM instrument (JEOL model 2100, JEOL Ltd., Tokyo, Japan).

The photocatalytic activities of PEDOT and PEDOT/ZnO nanocomposites were performed using MB dyes as degraded materials in quartz tubes under UV light and natural sunlight irradiation. FSL MW1-Y15 was used as the irradiation source (*λ* = 254 nm) located in a light-infiltrated chamber. According to the previous report [35], a 40-mL (1 × 10^-5^ M) dye solution (MB) was mixed with a desired amount of catalysts (0.4 mg/mL). Before irradiation, the suspension was stirred magnetically for 30 min in dark conditions until adsorption-desorption equilibrium was established, and then, the suspensions were irradiated by light sources with stirring. Under natural sunlight investigations, all experiments were done inside the laboratory in an open atmosphere in the month of June. The photodegradation efficiency (*R*,%) was calculated by the use of the equation *R* = [*C*_0_ - *C*/*C*_0_], where *C*_0_ represents the concentration of the dye before illumination and *C* denotes the concentration of the dye after a certain irradiation time, respectively.

## Results and discussion

### Fourier transform infrared spectroscopy

**Figure 1:**
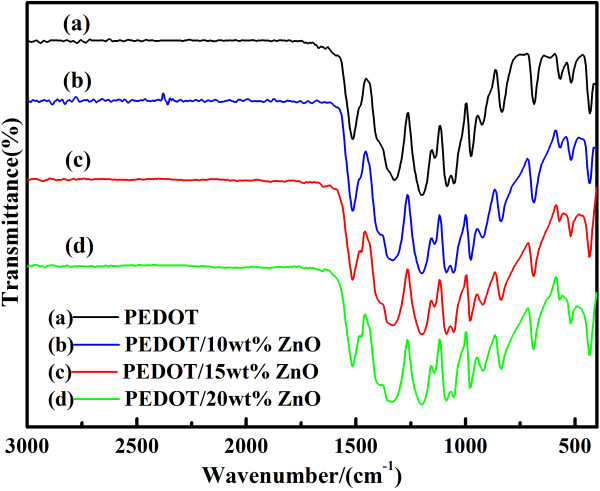
FTIR spectra of PEDOT and PEDOT/ZnO nanocomposites prepared from different weight percentages of nano-ZnO.

Figure 1 shows the FTIR spectra of PEDOT and PEDOT/ZnO nanocomposites. As can be seen in Figure 1, the main characteristic bands of composites are identical to that of PEDOT. The bands at approximately 1,510 and 1,310 cm^-1^ are assigned to the asymmetric stretching mode of C = C and the inter-ring stretching mode of C-C [36], respectively. The bands at approximately 1,200, 1,135, and 1,085 cm^-1^ are attributed to the C-O-C bending vibration in ethylenedioxy [37]. The bands at approximately 970, 915, 825, and 685 cm^-1^ are the characteristic bands of stretching vibrations of the C-S-C bond in the thiophene ring [38]. However, there are no characteristic peaks corresponding to the nano-ZnO in the composites, and this phenomenon is similar to the previously reported polyaniline/ZnO(30 wt%), in which there is no characteristic peak for ZnO [39].

### UV-vis spectra

**Figure 2:**
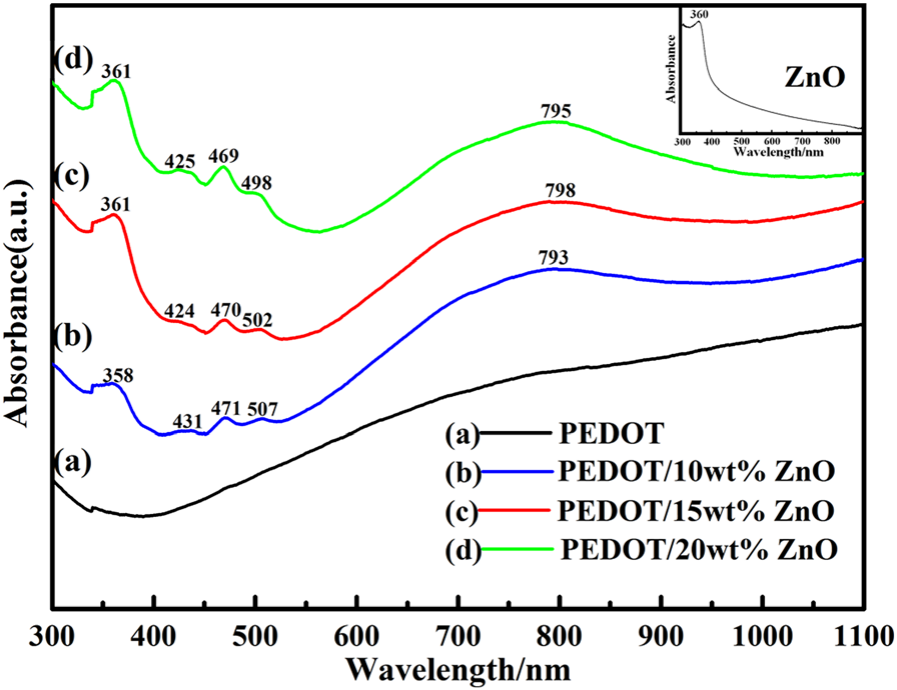
**UV-vis spectra of PEDOT and PEDOT/ZnO nanocomposites prepared from different weight percentages of nano-ZnO.** The inset shows the UV-vis spectra of nano-ZnO.

Figure 2 gives the UV-vis absorption spectra of PEDOT and PEDOT/ZnO nanocomposites in NMP. As shown in Figure 2, the UV-vis absorption spectrum of the pure PEDOT shows a broad absorption band in the vis-NIR, starting at approximately 500 nm and extending into the mid IR region, which corresponds to the polymer having a longer conjugation length with greater order, and it is presumably attributed to polaron and/or bipolaron bands [40]. Compared with the pure PEDOT, the strong characteristic bands of the PEDOT/ZnO nanocomposites locate at approximately 360, 425, 470, 503, and 795 nm, respectively. The strong absorption band at approximately 360 nm is corresponding to the nano-ZnO, which is in good agreement with the UV spectrum of the nano-ZnO (inserted image in Figure 2). The absorption bands at approximately 425, 470, and 505 nm can be considered as the absorption peaks arising from conjugated segments having different conjugation lengths, and they are assigned to the π→π* transition of the thiophene ring, while the appearance of the absorption band at approximately 795 nm is assigned to the polaron and/or bipolaron band, indicating a strong interaction between PEDOT and nano-ZnO [41,42]. Furthermore, the peak intensity ratio *I*_795_/*I*_360_ is 0.93 for PEDOT/15wt%ZnO, and it is 1.35 and 0.81 for PEDOT/20wt%ZnO and PEDOT/10wt%ZnO, respectively, which are quite in accordance with the variation of nano-ZnO content in composites.

### X**-**ray diffraction

**Figure 3:**
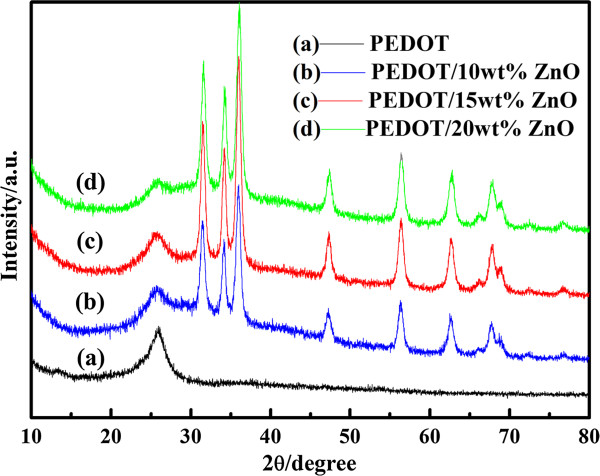
XRD patterns of PEDOT and PEDOT/ZnO nanocomposites prepared from different weight percentages of nano-ZnO.

Figure 3 shows the XRD patterns of PEDOT and PEDOT/ZnO nanocomposites. The XRD patterns of PEDOT shows only one characteristic peak at approximately 2*θ* = 25.9°, which are associated to the intermolecular π→π* stacking, corresponding to the (020) reflection of the polymer backbone [33,43,44]. In the case of composites, the diffraction peaks at 2*θ* = 31.5°, 34.2°, 35.9°, 47.3°, 56.3°, 62.6°, 66.2°, 67.7°, 68.9°, 72.5°, and 76.8° are associated to the (100), (002), (101), (102), (110), (103), (200), (112), (201), (004), and (202) planes of the nano-ZnO, which coincide with the peaks of the ZnO from other reports [30,45]. Therefore, the XRD patterns of composites suggest a successful incorporation of nano-ZnO in composites.

### Transmission electron microscopy

**Figure 4:**
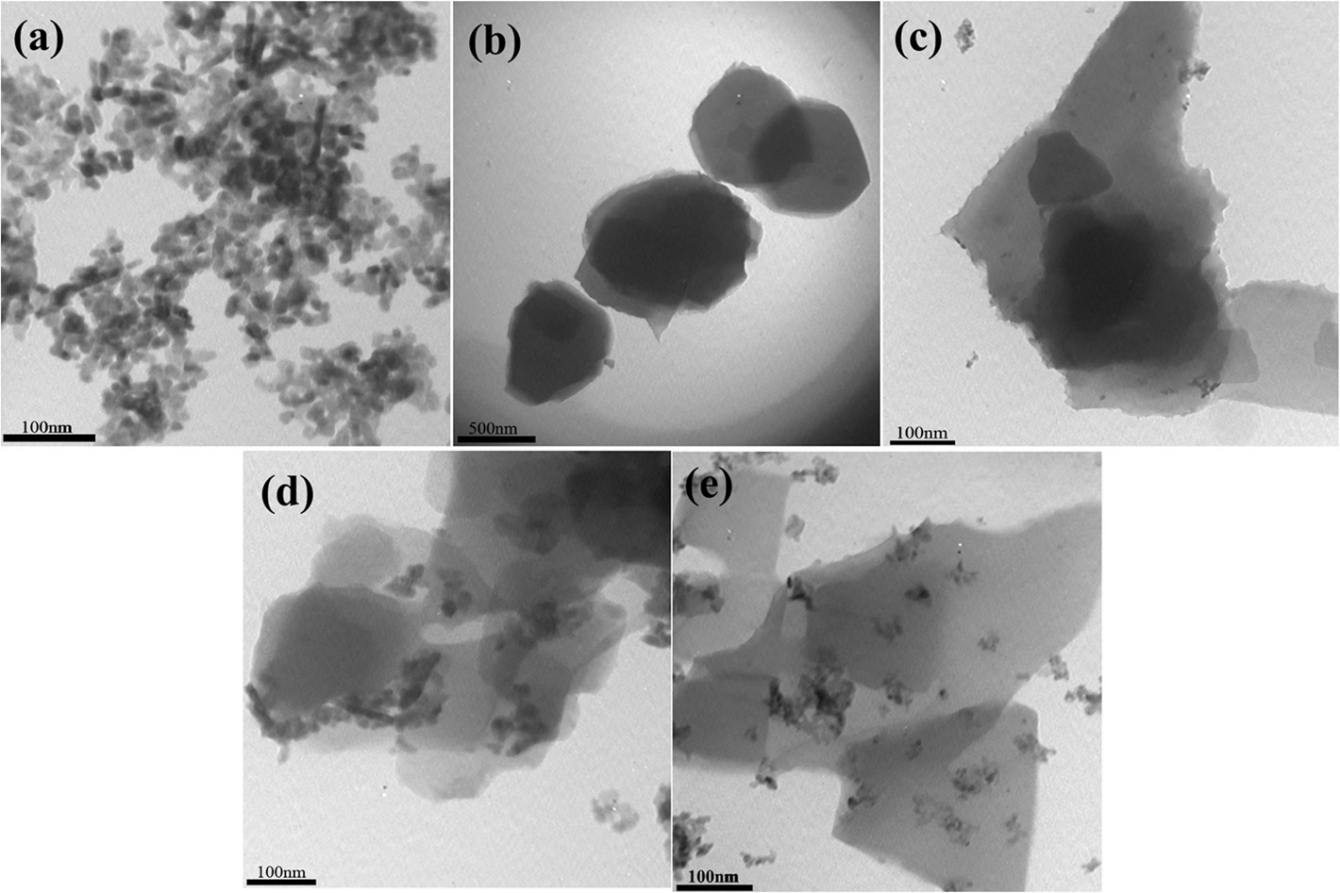
**TEM images of ZnO, PEDOT, and PEDOT/ZnO nanocomposites prepared from different weight percentages of ZnO. (a)** ZnO, **(b)** PEDOT, **(c)** PEDOT/10wt%ZnO, **(d)** PEDOT/15wt%ZnO, and **(e)** PEDOT/20wt%ZnO.

Figure 4 represents the TEM images of PEDOT and PEDOT/ZnO nanocomposites. The results from TEM indicate that the pure nano-ZnO consists of spherical-shaped particles with an average size of 50 nm. As seen from Figure 4a, PEDOT exhibits numerous shale-like morphology with layered structure. In the case of composites (Figure 4b,c), the shale-like PEDOT also occurred, and it is easy to identify the nano-ZnO. Furthermore, the very large aggregates of nano-ZnO were not observed.

### Photocatalytic activity

**Figure 5:**
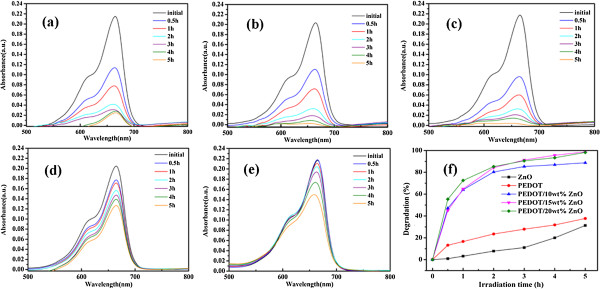
**UV-vis absorption spectra of MB dyes by photocatalysis for different irradiation times under UV light irradiation. (a)** PEDOT/10wt%ZnO, **(b)** PEDOT/15wt%ZnO, **(c)** PEDOT/20wt%ZnO, **(d)** pure PEDOT, **(e)** nano-ZnO, **(f)** degradation efficiency of the MB dyes (catalyst concentration 0.4 mg/mL, initial concentration of dyes 1 × 10^-5^ M).

**Figure 6:**
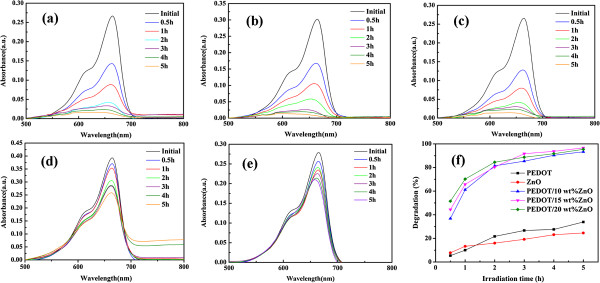
**UV-vis absorption spectra of MB dyes by photocatalysis for different irradiation times under natural sunlight irradiation. (a)** PEDOT/10wt%ZnO, **(b)** PEDOT/15wt%ZnO, **(c)** PEDOT/20wt%ZnO, **(d)** PEDOT, **(e)** nano-ZnO, **(f)** degradation efficiency of the MB dyes (catalyst concentration 0.4 mg/mL, initial concentration of dyes 1 × 10^-5^ M).

The photocatalytic degradation of the MB dye in the presence of the PEDOT/ZnO nanocomposite as catalyst under UV and natural sunlight sources at different irradiation times was investigated. For comparison, the degradation efficiency of the MB dye by pure PEDOT and nano-ZnO under both light sources as well as the adsorption mechanisms of the MB dye by ZnO particles in dark condition and under UV light irradiation without catalysis was also investigated. As depicted in Figures 5 and 6, the decrease of the absorption band intensities of the MB dye indicates that the MB dye can be degraded by PEDOT/ZnO nanocomposites, pure PEDOT, and nano-ZnO under both UV and natural sunlight. Moreover, under UV light source, the degradation efficiency of MB is 88.7%, 98.7%, and 98.2% for PEDOT/10wt%ZnO, PEDOT/15wt%ZnO, and PEDOT/20wt%ZnO nanocomposites, respectively, and under natural sunlight source, the degradation efficiency of MB is 93.3%, 96.6%, and 95.4% for PEDOT/10wt%ZnO, PEDOT/15wt%ZnO, and PEDOT/20wt%ZnO nanocomposites, respectively. However, in the case of pure PEDOT and nano-ZnO, the degradation efficiencies of the MB dye are 37.7% and 31.3% under UV light for PEDOT and nano-ZnO, respectively, while the degradation efficiencies of the MB dye are 33.9% and 24.3% under natural sunlight for PEDOT and nano-ZnO, respectively.

**Figure 7:**
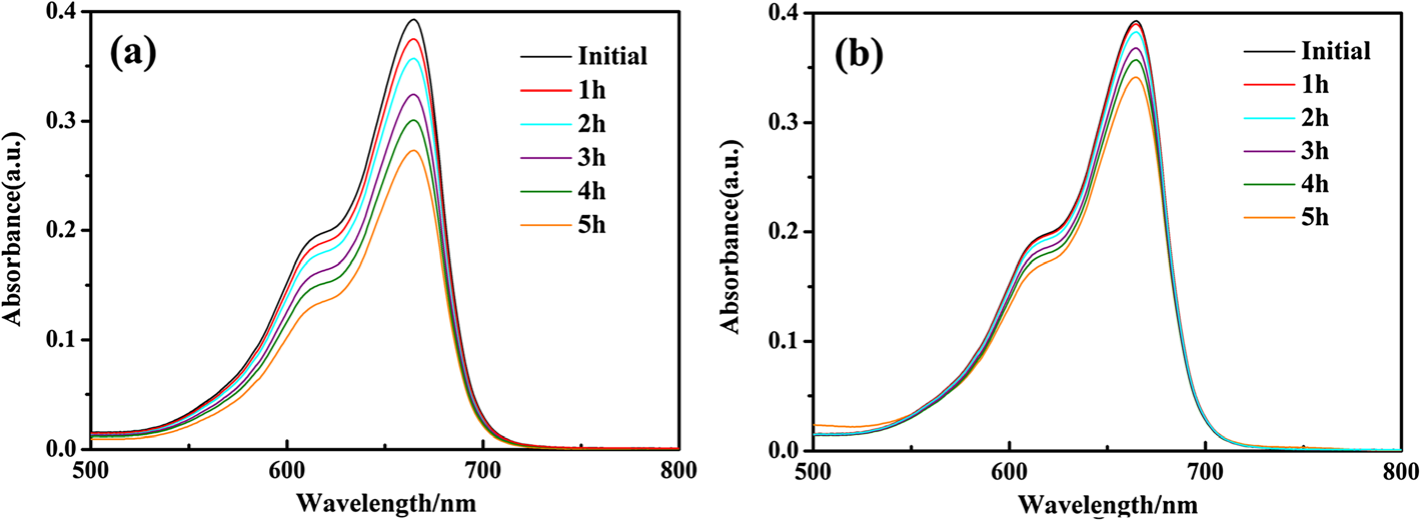
**UV-vis absorption spectra. (a)** MB dye without catalysis under UV light irradiation. **(b)** MB dye by ZnO catalysis under dark condition.

As shown in Figure 7, the adsorption of the MB dye is 27% under UV light irradiation without catalysis and 17% in dark condition by ZnO particles in 5 h, which suggests that the adsorption of the MB dye under both conditions is very low. All these results revealed that the degradation efficiencies of pure PEDOT and nano-ZnO are lower than those of PEDOT/ZnO nanocomposites under the same conditions. Furthermore, the photocatalytic activity of the composites decreases with the increasing amount of nano-ZnO. Therefore, it can be concluded that the synergic effects between pure PEDOT and nano-ZnO can play an important role to increase the photocatalytic activity of the composites. It should be noticed that the degradation efficiency of MB by PEDOT/ZnO is higher than that (94% after 6 h) of MB by polyaniline/ZnO nanocomposite [35] and higher than that (88.5% in 10 h) of methyl orange (MG) by poly(3-hexylthiophene)/TiO_2_ nanocomposites under sunlight irradiation [46].

**Figure 8:**
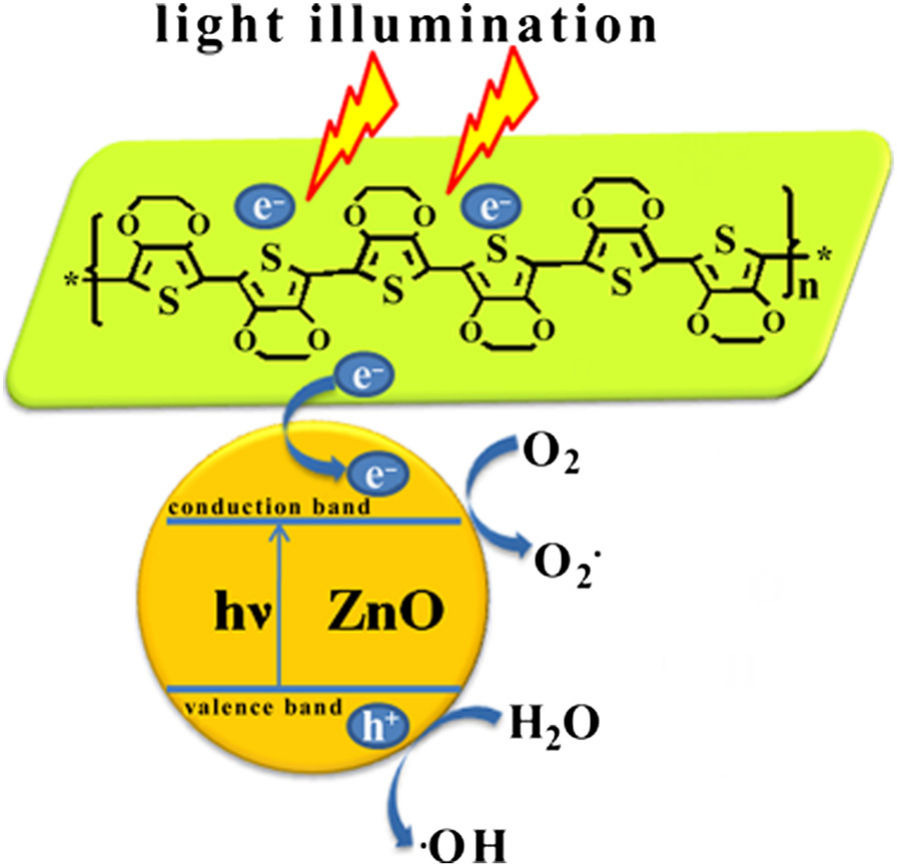
A schematic illustration of the photocatalytic activity of PEDOT/ZnO nanocomposites.

Figure 8 shows the schematic mechanism of MB dye degradation to explain the photocatalytic activity of the PEDOT/ZnO nanocomposite catalyst under visible light. According to the previous report, light illumination on the nanocomposite catalyst can cause the generation of electron (e^-^) in the conduction band and holes (h^+^) in the valence band [47]. In addition, the pure PEDOT can absorb the visible light and produces an electron (e^-^) that transfers to the conduction band of nano-ZnO, which will lead to an enhancement in charge separation and the formation of oxyradicals (O_2_, HO_2_, OH) [47,48]. Consequently, the high amount of oxyradicals (O_2_, HO_2_, OH) results in high MB degradation under visible light.

## Conclusions

The PEDOT/ZnO nanocomposites in powder form with the content of ZnO varying from 10 to 20 wt% were prepared by a simple solid-state heating method. The results confirmed that the ZnO nanoparticles were successfully incorporated in the PEDOT matrix through solid-state polymerization, and there was a strong interaction between PEDOT and nano-ZnO. Compared with the existing methods, the method demonstrated here is facile but effective and could be readily used for a large-scale preparation of this type of composites. Furthermore, the PEDOT/ZnO nanocomposite is in powder form, which can expand its use in electro-optical devices. The photocatalytic results showed that the incorporation of ZnO nanoparticles to the composites can enhance the photocatalytic efficiency under UV light and natural sunlight irradiation, which was attributed to the efficiently high charge separation of electron and hole pairs in this type of composite materials. This indicates a potential application of PEDOT/ZnO nanocomposites for dye UV-vis photodegradation.

## Competing interests

The authors declare that they have no competing interests.

## Authors’ contributions

TA conceived the study, carried out the data analysis, and drafted the manuscript. AA carried out the sample preparation and the experimental measure. RJ participated in the study of material structures and the data analysis. YO and YZ coordinated the research and revised the manuscript. All authors read and approved the final version of the manuscript.
